# The relationship between social mobility and mortality: analysis of intercensal data (1991, 2001, and 2011)

**DOI:** 10.1093/eurpub/ckag097

**Published:** 2026-06-12

**Authors:** Michael Rosato, Rachel McCarter, Gerard Leavey

**Affiliations:** The Bamford Centre for Mental Health & Wellbeing, Ulster University, Belfast, United Kingdom; The Bamford Centre for Mental Health & Wellbeing, Ulster University, Belfast, United Kingdom; The Bamford Centre for Mental Health & Wellbeing, Ulster University, Belfast, United Kingdom

## Abstract

Persisting social disadvantage is associated with poor health and higher mortality. Increasing social mobility is, therefore, a major policy goal in the United Kingdom and other Western nations. However, the extent to which mortality is attenuated by upward social mobility is unclear, evidence from lifecourse studies suggests the persisting effects of early disadvantage. We sought to examine the relationship between intercensal social mobility and all-cause mortality. Census returns from three waves of the Northern Ireland Longitudinal Study (1991 *n* = 439 553; 2001 *n* = 446 573; 2011 *n* = 480 031) linked with mortality data. Using census data on ‘educational attainment’, ‘occupational social class’, ‘housing tenure’, and ‘household car access’ we derived an index of individual and household-level disadvantage and examined intercensal social mobility and all-cause mortality between 1991–2001 and 2001–2011. At each census point, all-cause mortality was positively associated with increasing social disadvantage. Relative to the least disadvantaged at each census, populations in the most disadvantaged areas recorded (hazard ratios = 3.23 (95% CI 2.49, 4.19); HR = 5.09 (4.21, 6.17), and HR = 5.30 (4.56, 6.16)) for 1991, 2001, and 2011, respectively. Compared with the socially static groups, those with generally downward social mobility showed an excess mortality, while mortality in the upwardly social mobility groups was generally lower than (or similar to) those in more socially static groups. Perhaps predictably, all-cause mortality was strongly associated with downward social mobility across time periods. Of greater concern, the Hazards associated with downward mobility appear to intensify over time. Moreover, increased social mobility appears to have little positive impact on mortality.

## Introduction

Socio-economic circumstance (SEC) (usually measured by income, occupation and educational attainment) influences health and social outcomes by directly determining life chances and access to goods and services crucial across the life-course. The household risk factors associated with poverty (e.g. poor diet, inadequate heating and lighting, and the fabric and design of dwellings) are often situated within an array of environmental health risk factors which include exposure to poor air quality, and limited access to community assets such as parks and health-promoting spaces. Evidence of a clear social relationship between poor health and lower SEC has been observed internationally for most health and social outcomes [1, 2]. Evidence also suggests mortality risks increase as social disadvantage increases [3]. Closing this ‘social inequity’ gap through policies designed to promote upward social mobility–defined as (lasting) change in improved social circumstances between or within generations—is seen as a governmental policy priority, certainly in Western developed economies.

However, the evidence for a consistent relationship between social mobility and health outcomes is unclear Blane et al [4], using the Office for National Statistics Longitudinal Study (ONS-LS) for England and Wales (E&W) found that, relatively socially mobile individuals (compared with more ‘socially stable’ counterparts) found an inverse relationship between upward mobility and lower mortality, suggesting, that socioeconomic mortality differentials can persist with increasing age. Bartley and Plewis [5] suggest that upward mobility may be linked with poorer health outcomes, possibly due to stress arising from an incongruence between current and past socio-economic conditions [6]. However, it has been argued that such links between class at birth and health outcomes are overly deterministic, failing to account for potentially intervening social mechanisms. Thus, the development of social capital in deprived neighbourhoods may attenuate the negative effects of poverty on health [7–9], rather than childhood determinants which appear to lock individuals into poor social and health outcomes over the life course. Other evidence suggests that poor health associated with living in disadvantaged neighbourhoods may be exacerbated by ethnicity and low education attainment, amplifying social inequalities in health [10]. Nevertheless, the evidence for the health benefits attached to social mobility is unclear.

The uncertainty about the relationship between social mobility and health outcomes may be explained by variability in the measurement of both SEC and social mobility. Therefore, while area-level deprivation may persist over time in a particular geographical area, there is a constant movement of people in and out of these areas [11, 12]. In the UK, a net population shift over time has been noted from deprived to more affluent areas [13–15]. Furthermore, any association between social mobility and mortality may be obscured by the ecological fallacy (area-level measurement may not apply to everyone in that area)—therefore, individuals with a higher individual-level socioeconomic position resident in more deprived areas may record lower mortality relative to the average for the area [16].

Moreover, conceptual differences between the measurement of social mobility in absolute and relative terms are also noted [17], whereby absolute mobility evaluates an individual’s social class position in relation to their parental social class, and this measure appears to be linked to an individual’s lived experiences [18, 19]. Finally, while a considerable increase in absolute social mobility in the United Kingdom (UK) in the second half of the 20th century [20] this appears to have stalled in the more recent past [18]. Because of the importance of these structural phenomena—e.g. persisting deprivation and governmental assignment of resources—in the conceptualization of ongoing societal cohesion, it is important to periodically examine both the extent of social mobility and what of its effects can be measured. An understanding of the current deprivation indices assist in assigning resources by local and central government [21–23]. Frequency of residential relocation is associated with both socio-economic adversity and health [24].

## Aim

Using census-derived databases from Northern Ireland (NI): to examine the relationship between persisting socio-economic disadvantage, social mobility (measured at two time points between 1991–2001 and 2001–2011) and all-cause mortality subsequent to each of these periods.

## Method

This study was undertaken through the ‘Administrative Data Research Centre NI’ (ADRC-NI), a UK initiative to extend the use of administrative or routine data sources in research [25]. It uses the ‘Northern Ireland Longitudinal Study’ (NILS)—while this is described in detail elsewhere [26], briefly it is a representative 28% sample of the NI census-enumerated population drawn from each of census years 1991, 2001, and 2011, data linked, managed and maintained by the ‘Northern Ireland Statistics and Research Agency’ (NISRA), an executive agency of the NI Department of Finance [27]. The method for selection into NILS ensures that the sampled cohort remains consistent through time, and the electronic linking of the three census points ensures longitudinality in the individual records from 1991 to 2011. Census data were linked with death records (1991–2015) from the NI General Register Office.

‘Socio-demographic characteristics’ were separately derived for each of the three census points—1991, 2001, and 2011: age group (in 5-year age bands from 25 to 74 years); gender (male/female); locale of residence (summarised as rural, intermediate, or urban); marital status (single, married, or separated/divorced/widowed); educational attainment (degree level, intermediate level, or no qualifications); occupational social class (three groups—professional, intermediate, and routine occupations); housing tenure (owner occupation, renting); and household car access (two or more cars, one car only, no car access). Ethnicity may have important effects on outcomes but unfortunately these data were not collected for the 1991 Northern Ireland Census as in other parts of the UK.

*Socio-economic circumstances (SEC)*: Education, Occupational Group, Housing tenure, and Car access (broadly representing socio-economic circumstance) were formulated as ranging from most advantaged (scored as zero) to least advantaged (scored as one or more), combined (for each census separately) and accumulated to form a set of three indices (each ranging from 0 to 7) representing individual and household-level social disadvantage [28].

Our outcome variable was ‘all-cause mortality’ in the 5 years following each census: given that the contextual socio-economic data is collected at the associated census point only, restricting the follow-up period to the subsequent 5 years allows that the (essentially dynamic) contextual data around socio-economic circumstance will not diverge substantially from its initial recorded values.

## Analysis

Because of the confidential nature of Census data, the analysis process is strictly controlled and designed to ensure data privacy: all researchers accessing data must be accredited; with research carried out in a secure physical setting using data de-identified prior to access; and all outputs scrutinized by the data custodians for inadvertent breaches of privacy prior to release. Statistical analyses were performed using Stata/SE version 17.0 [29]. The analysis comprised NILS members aged 25 to 74 years at each census point, resident in private households, and normally resident in NI. Frequencies and related percentages are presented for all included variables. For each census cohort separately the effects of disadvantage on all-cause mortality are measured using both age-standardized rates (ASR, per 100 000 population) and Cox Proportional Hazards (PH) models examined ‘all-cause mortality’. As is usual practice in deriving ASRs, 5-year age groups were used. Finally, Cox PH models examined intercensal ‘social mobility’ between both the 1991 and 2001 Census and (separately) the 2001 and 2011 Census. Here the ‘mobility’ analysis is stratified by each level of disadvantage at the point of origin–with the mobility trajectory measured at the point of destination. In these analyses the reference category is always those persons who experience no census mobility.

## Results

Supplementary Fig. S1 shows the age-structure of the three NILS cohorts at each of the three census points: the 1991 cohort peaks at 25–29 years, before declining slowly to ages 70–74 years; the 2001 cohort shows a similar but smaller peak at ages 35–39; and finally, the 2011 cohort peaks at ages 45–49 years, before declining, though more slowly than with the earlier cohorts. This pattern is typical of a population structure ageing over time–suggesting a gradual evening out of the 5-year age groups, with relatively fewer younger and relatively more older people.

The sex distribution, 52% female and 48% male, remains stable over the 20-year study period (Table 1). Across each census: the proportions in ‘professional occupations’ is constant (31%–32%); those in ‘routine occupations’ reduced by 10% (27%–16% between 1991 and 2011); with those in ‘intermediate occupations’ increasing from 42% to 52%; those with ‘degree-level education’ increased from 8% to 32%, and those recording ‘no qualifications’ fell from 69% to 28%; households with ‘two or more cars’ increased from 27% to 50%, and those with ‘no car access’ reduced from 25% to 14%; and finally, housing tenure—‘owner occupation’ rose from 70% to 79% between 1991 and 2001, before reducing slightly to 76% in 2011. The ‘index of household-level disadvantage’ is derived from the four broadly socio-economic characteristics outlined above: here the ‘least deprived’ groups (levels 0–2) record proportional intercensal increases (e.g. level 2 numbers increased from 15% in 1991 through 18%–22% in 2011); level three represents a pivot point of relative intercensal stability; and the ‘more disadvantaged’ groups (4–7) recording decreasing intercensal proportions (e.g. proportions in the most disadvantaged group decreased in size from 5.4% in 1991 to 1.75% in 2011).

**Table 1.** Social and economic demography associated with the Northern Ireland Longitudinal Study—derived from the census-enumerated population (aged 25–74) for the census years 1991, 2001, and 2011.^a^

		1991	2001	2011
Gender	Male	48.3 (115 424)	48.0 (123 869)	48.1 (139 347)
	Female	51.7 (123 704)	52.0 (134 391)	51.9 (150.346)
Social class	Professional	31.0 (52 579)	30.6 (73 259)	32.3 (86 292)
	Intermediate	42.2 (71.640)	50.9 (121 831)	52.3 (139 941)
	Routine	26.8 (45 467)	18.5 (44 367)	15.5 (41 348)
Education	Degree or higher	7.8 (18 722)	16.7 (42 972)	31.7 (91 621)
	Intermediate level	23.1 (55 134)	35.9 (92 274)	40.5 (117 166)
	No qualifications stated	69.11 (165 272)	47.4 (121 995)	27.9 (80 738)
Housing tenure	Owner occupier	69.7 (165 148)	78.5 (201 461)	75.8 (218.511)
	Renting	30.3 (71 906)	21.5 (55 241)	24.2 (69 751)
Household car access	Two or more cars	27.0 (63 917)	41.3 (106 664)	50.1 (145 065)
	One car only	48.2 (114 245)	43.5 (112 421)	36.5 (105,64)
	No car access	24.8 (58 892)	15.2 (39 175)	13.5 (38 988)
Index of individual-level disadvantage	0: least	4.8 (8065)	8.3 (19 806)	13.8 (36 889)
	1:	9.1 (15 408)	12.7 (30 320)	15.7 (42 020)
	2:	15.0 (25 332)	17.8 (42.444)	22.4 (59 887)
	3:	20.2 (34 106)	21.5 (51 241)	19.1 (51 013)
	4:	20.2 (34 220)	18.1 (43 270)	13.9 (37 150)
	5:	15.6 (26 445)	12.2 (29 164)	8.7 (23.173)
	6:	9.7 (16.362)	6.7 (15.872)	4.5 (12 101)
	7: most	5.4 (9209)	2.8 (6672)	1.75 (4659)

a Data represents proportions (and numbers).

Generally, for each census cohort (Table 2) ASRs associated with all-cause mortality increase with levels of ‘rising disadvantage’: e.g. the 2001 cohort recorded ASR = 243 (95% Confidence intervals = 193, 293) and ASR = 1244 (1130, 1357) for the least and most disadvantaged groups respectively. Furthermore, these results suggest that this rising disadvantage/mortality relationship is deepening over time: looking across the cohorts persons in the ‘least disadvantaged’ groups (levels 0–2) record ASRs reducing over time, while those in the ‘more disadvantaged’ groups (levels 4–7) record ASRs increasing over time: e.g. level 2 records ASR = 509 (95%CI = 435, 583), ASR = 450 (411, 489) and ASR = 386 (359, 414) for the 1991, 2001, and 2011 cohorts respectively; while level 5 records ASR = 668 (615, 722), ASR = 754 (713, 796), and ASR = 775 (726, 823) for the 1991, 2001, and 2011 cohorts, respectively. This trend is reflected also in the associated Hazard Ratios (HRs): while all three census points record mortality rising with ‘increasing disadvantage’ this rise gets steeper with each passing census—e.g. compared with the ‘least disadvantaged’ at each census those ‘most disadvantaged’ record increasing magnitudes HR = 3.23 (95%CI = 2.49, 4.14); HR = 5.09 (4.21, 6.17); and HR = 5.30 (4.56, 6.16) for 1991, 2001, and 2011 respectively.

**Table 2.** All-cause mortality (for persons aged 25–74 years) in Northern Ireland for years 1991–1995, 2001–2005 and 20011–2015, by level of social disadvantage, derived separately at census years 1991, 2001 and 2011.^a^

Index of individual-level disadvantage	1991		2001		2011	
	Age/sex standardized rate (95% CI)	Social disadvantage (models adjusted for age/sex) HR (95% CI)	Age/sex standardized rate (95% CI)	Social disadvantage (models adjusted for age/sex) HR (95% CI)	Age/sex standardized rate (95% CI)	Social disadvantage (models adjusted for age/sex) HR (95% CI)
0: least	300 (206, 394)	1.00	243 (193, 293)	1.00	251 (221, 281)	1.00
1:	514 (385, 642)	1.30 (0.97, 1.72)	346 (305, 387)	1.43 (1.17, 1.75)***	312 (284, 340)	1.28 (1.07, 1.47)**
2:	509 (435, 583)	1.54 (1.18, 2.00)**	450 (411, 489)	1.81 (1.50, 2.19)***	386 (359, 414)	1.57 (1.38, 1.79)***
3:	575 (518, 632)	1.76 (1.37, 2.27)***	487 (458, 517)	2.05 (1.71 (2.45)***	443 (417, 470)	1.87 (1.65, 2.12)***
4:	589 (519, 611)	1.81 (1.40, 2.32)***	603 (572, 634)	2..56 (2.14, 3.05)***	599 (565, 633)	2.50 (2.20, 2.83)***
5:	668 (615, 722)	2.11 (1.64, 2.72)***	754 (713, 796)	3.19 (2.67, 3.81)***	775 (726, 823)	3.21 (2.82, 3.64)***
6:	908 (834, 981)	2.87 (2.22, 3.69)***	1070 (1001, 1187)	4.34 (3.63, 5.20)***	1024 (947, 1100)	4.12 (3.61, 4.71)***
7: most	1025 (926, 1124)	3.23 (2.49, 4.19)***	1244 (1130, 1357)	5.09 (4.21, 6.17)***	1320 (1180, 1460)	5.30 (4.56, 6.16)***

a Data collected from the census-enumerated populations aged 25–74 at each census, and represents (i) age/sex standardized all-cause mortality rates (and 95% CIs) per 100 000 of the population; and (ii) all-cause mortality hazard ratios (and 95% CIs). ** <0.005; ***<0.0005.

Tables 3 and 4 shows HRs for all-cause mortality by social mobility between 1991–2001 (mortality between 2001 and 2005), and 2001–2011 (mortality between 2011 and 2015). As noted above each comprises analyses stratified by level of disadvantage at the earlier census of origin—Table 3 shows the effects of disadvantage in 2001, stratified by level of disadvantage in 1991; and similarly, Table 4 shows the effects of disadvantage in 2011, stratified by level of 2001 disadvantage. At each analysis therefore, the reference group for comparison is that corresponding to level of disadvantage at the point of origin (and the socially stable point in the mobility matrix). For mobility between 1991 and 2001 (Table 3), in relation to the static (or ‘non-mobile’) reference groups, ‘downward mobility’ was generally associated with excess mortality (though not all are statistically significant)—e.g. compared with level 3 persons recording ‘no mobility’ between 1991 and 2001, those ‘downwardly mobile’ recorded HR = 1.28 (95%CI = 1.06, 1.54); HR = 1.71 (1.31, 2.24) and HR = 2.61 (1.49, 4.58), and HR = 3.04 (0.97, 9.50); with ‘upward mobility’ associated with likelihoods similar to the reference group—e.g. HR = 0.99 (0.79, 1.25); HR = 1.06 (0.78, 1.45); and HR = 0.99 (0.53, 1.91). For mobility between 2001 and 2011 (Table 4)—generally, patterns noted in Table 3 are replicated: again compared with level 3 persons recording ‘no mobility’ between 2001 and 2011, those ‘downwardly mobile’ recorded HR = 1.23 (1.05, 1.45); HR = 1.75 (1.40, 2.20); HR = 1.51 (0.94, 2.42); and HR = 1.96 (2.18, 7.22); with ‘upward mobility’ associated with likelihoods similar to or lower than the reference group—e.g. HR = 0.84 (0.71, 1.00); HR = 0.77 (0.60, 0.99); and HR = 0.59: (0.32, 1.07). That the HRs associated with ‘downward mobility’ mainly record likelihoods higher than with the earlier decade suggests an intercensal intensification of the effect on mortality by ‘downward mobility’.

**Table 3.** Social mobility (1991–2001): all-cause mortality in Northern Ireland (2001–2005) for persons aged 35–74 at the 2001 Census—individual/household disadvantage at point of origin (1991) by disadvantage level at destination (2001).^a^

	Level of disadvantage: origin (1991)							
Level of disadvantage: destination (2001)	0: least disadvantaged HR (95% CI)	1: HR (95% CI)	2: HR (95% CI)	3: HR (95% CI)	4: HR (95% CI)	5: HR (95% CI)	6: HR (95% CI)	7: most disadvantaged HR (95% CI)
0: least	1.00	0.54 (0.35, 0.83)**	0.76 (0.48, 1.22)	1.01 (0.53, 1.91)	1.12 (0.46, 2.71)	0.90 (0.22, 3.62)	3.21 (0.79, 13.1)	N/A
1	1.63 (1.03, 2.58)*	1.00	0.78 (0.58, 1.05)	1.06 (0.78, 1.45)	0.55 (0.31, 0.98)*	1.04 (0.53, 2.03)	0.25 (0.04, 1.79)	0.50 (0.07, 3.56)
2	*2.00 (0.84, 4.71)*	*1.27 (0.92, 1.76)*	1.00	0.99 (0.79, 1.25)	1.11 (0.87. 1.41)	0.80 (0.54, 1.20)	0.35 (0.13, 0.93)*	0.43 (0.11, 1.74)
3	*0.80 (0.11, 5.82)*	*0.97 (0.55, 1.70)*	*1.04 (0.82, 1.31)*	1.00	1.01 (0.85, 1.19)	1.03 (0.83, 1.28)	0.70 (0.48, 1.02)	0.41 (0.19, 0.88)*
4	*1.18 (0.16, 8.57)*	2.22 (1.16, 4.28)*	*1.17 (0.86, 1.60)*	1.28 (1.06, 1.54)*	1.00	0.95 (0.80, 1.13)	0.68 (0.52, 0.87)**	0.87 (0.60, 1.28)
5	10.9 (2.64, 45.4)***	*1.78 (0.44, 7.27)*	*1.23 (0.65, 2.34)*	1.71 (1.31, 2.24)***	*1.12 (0.94, 1.34)*	1.00	0.80 (0.65, 0.99)*	0.71 (0.53, 0.95)*
6	12.0 (1.58, 91.2)*	*2.87 (0.40, 20.6)*	*2.22 (0.71, 6.98)*	2.61 (1.49, 4.58)***	*1.10 (0.75, 1.62)*	1.30 (1.05, 1.63)*	1.00	0.92 (0.72, 1.17)
7: most	*N/A*	*N/A*	*6.21 (0.87, 44.5)*	*3.04 (0.97, 9.50)*	2.87 (1.53, 5.38)***	1.88 (1.26, 2.82)**	*1.30 (1.00, 1.70)*	1.00

a All models are adjusted for age and gender and data represents hazard ratios (HRs) and 95% confidence intervals. *<0.05; **<0.005; ***<0.0005.

**Table 4.** Social mobility (2001–2011): all-cause mortality in Northern Ireland (2011–2015) for persons aged 35–74 at the 2011 Census—individual/household disadvantage at point of origin (2001) by disadvantage level at destination (2011).^a^

	Level of disadvantage: origin (2001)							
Level of disadvantage: destination (2011)	0: least disadvantaged # HR (95% CI)	1: HR (95% CI)	2: HR (95% CI)	3: HR (95% CI)	4: HR (95% CI)	5: HR (95% CI)	6: HR (95% CI)	7: most disadvantaged HR (95% CI)
0: least	1.00	0.80 (0.61, 1.05)	0.85 (0.60, 1.20)	0.59 (0.32, 1.07)	0.65 (0.29, 1.45)	0.20 (0.03, 1.45)	n/a	n/a
1	*1.02 (0.73, 1.43)*	1.00	1.00 (0.80, 1.24)	0.77 (0.60, 0.99)*	0.73 (0.50. 1.05)	1.07 (0.63, 1.84)	0.57 (0.18, 1.80)	0.70 (0.10, 5.09)
2	*1.43 (0.84, 2.44)*	*1.29 (1.01, 1.67)* *	1.00	0.84 (0.71, 1.00)	0.86 (0.70, 1.07)	0.75 (0.52, 1.08)	0.60 (0.30, 1.22)	n/a
3	*2.56 (1.30, 5.02)* *	*1.37 (0.93, 2.02)*	*1.05 (0.84, 1.30)*	1.00	0.84 (0.72, 0.99)*	0.84 (0.67, 1.06)	0.84 (0.56, 1.26)	0.92 (0.43, 1.99)
4	*0.59 (0.08, 4.23)*	*2.11 (1.26, 3.54)* **	*1.77 (1.35, 2.31)* ***	*1.23 (1.05, 1.45)* *	1.00	0.87 (0.74, 1.04)	0.96 (0.73, 1.26)	1.07 (0.67, 1.69)
5	*3.21 (0.79, 13.0)*	*3.07 (1.44, 6.57)* **	*1.90 (1.21, 2.97)* **	*1.75 (1.40, 2.20)* ***	*1.43 (1.22, 1.68)* ***	1.00	0.92 (0.74, 1.14)	0.51 (0.32, 0.82)**
6	*12.2 (3.01, 49.3)* ***	*2.64 (0.65, 10.7)*	*3.49 (1.72, 7.07)* ***	*1.51 (0.94, 2.42)*	*1.67 (1.30, 2.16)* ***	*1.26 (1.04, 1.52)* *	1.00	0.89 (0.67, 1.18)
7: most	*n/a*	*18.4 (5.85, 57.9)* ***	*3.62 (0.90, 14.6)*	*3.96 (2.18, 7.22)* ***	*2.18 (1.32, 3.60)* **	*1.63 (1.19, 2.24)* **	*1.45 (1.15, 1.83)* **	1.00

a All models are adjusted for age and gender and data represents hazard ratios (HRs) and 95% confidence intervals. *<0.05; **<0.005; ***<0.0005.

## Discussion

This is the first longitudinal study that combines three waves of national census data to examine the link between social mobility and mortality. These findings reveal an ageing population over the 20-year study period, but also one that is increasingly educated and more affluent, as shown in the relative shift of people from lower paid ‘routine’ occupational groups to relatively more affluent ‘intermediate’ occupations, and stability in the intercensal proportions of those in ‘professional’ roles. This patterning indicates evolution in the types of work available and the education levels necessary to attain them, but also recognizes a ceiling to mobility in terms of available professional occupations and attendant affluence. However, the findings also note the persistence of disadvantage which, while reducing substantially over the time period, remains evident. In the mortality analysis, as with other studies, we note a consistent social gradient with rising mortality in line with level of disadvantage. Importantly, in relation to social mobility, we found that while upward mobility has little impact on all-cause mortality relative to those whose social circumstances remained stable at each census, downward mobility is associated with increased mortality which intensified over the census time points.

Previous evidence suggests that upward mobility may confer a health reward, relative to those who remain in disadvantage, possibly through better health care access and greater opportunities for healthier lifestyles [30, 31]. Alternatively, what appears more likely (and supported by our findings) is that the health of upwardly mobile individuals may improve slightly but falls short of those with consistently high socio-economic circumstances. Thus, consonant with a life course framework [32], the pathways to chronic illnesses and poor health, tend to be fixed earlier in the life-course and may also be determined across generations. Early life experiences, including low birth-weight, have latent effects that may lead a range of chronic disorders such as cardiovascular disease. Indeed, Ben-Schlomo and Kuh [32] provide a model of childhood adversity characterized by poor diet and worse educational opportunities. Risk factors at different life stages may also accumulate over time because of ‘chains of risk’ where one adverse (beneficial) exposure or experience tends to lead to another, and so on. For example, unemployment can lead to financial insecurity, which in turn may increase the likelihood of marital conflict and divorce. While these ‘links’ are probabilistic rather than deterministic, they are likely to be sequential [33, 34].

### Strengths and limitations

This study is based on census information and therefore has all the strengths and limitations associated with such data. Thus, the sample is large and representative, with no inherent bias in the sampling process but derived from information collected for processes other than research. For example, while the derived ‘index of individual/household disadvantage’ can adequately represent a summation of socio-economic deficits it is not a validated tool, and any nuance associated specifically within each of its component indicators is lost to the analysis. Nevertheless, we have devised a robust approach, which captures important elements of social and demographic change over the previous 20 years. Finally, for the mobility analysis—while each cohort selected is aged 25 to 74 years at its census entry-point, the mortality is measured from the respective mobility destination end-points (and therefore deaths between 35 to 74 years), reducing the generalizability of these results.

Migration to Northern Ireland has historically been low, growing only modestly over the 30-year period under consideration here (1991–2011) compared to other UK and European nations. Low migration may be due to a combination of high unemployment and civil unrest. Additionally: the data collected at the three Censuses on ‘ethnicity/country of birth’ has changed oner the three time-points, making a robust and sensitive indicator difficult to devise. Thus, the 1991, census did not collect ethnicity data. As this in-migrant population to Northern Ireland has grown over the period 2011–2021, a more robust analysis of ethnicity/country of birth may be more feasible. In the case of our analysis, we felt analyses specifically related to ‘ethnicity/country of birth’ would not have been robust.

For this study, we focussed on the on the covariate effects on ‘all-cause mortality’ over the three time-periods.; an examination and reporting of selected cause-specific mortality in this paper could have been unmanageable. The data custodians for the Northern Ireland Longitudinal Study (NISRA) require researchers to be quite specific when designing individual research projects as a means of controlling data shared with researchers, requiring the submission of a new project application to do so. We will address this in future studies.

## Supplementary Material

ckag097_Supplementary_Data

## Data Availability

The data analysed in this study are available through the *Northern Ireland Statistics and Research Agency* (NISRA) and can be examined on application within the secure setting. Key points Upward social mobility appears to facilitate a longer life, relative to those who remain in disadvantage. The health of upwardly mobile individuals may improve slightly but not equal to those with those whose socio-economic circumstances are consistently high. Consonant with a life course framework, poor health and mortality may be determined earlier in the life-course.
